# Inflammatory CNS lesion and interstitial pneumonia as manifestations of pediatric Behçet’s disease

**DOI:** 10.1186/1546-0096-10-S1-A94

**Published:** 2012-07-13

**Authors:** Ana Beatriz Vargas, Cynthia T França, Blanca ERG Bica

**Affiliations:** 1 Hospital Universitário Clementino Fraga Filho - Universidade Federal do Rio de Janeiro, Rio de Janeiro, Brazil; 2 Hospital Universitário Clementino Fraga Filho - Universidade Federal do Rio de Janeiro, Niteroi, Brazil

## Purpose

Behçet’s disease is clinically heterogeneous. The little available pediatric data suggest that the most common CNS presentation is vasculitis without parenchyma compromise and the most common thoracic manifestation is pulmonary artery aneurism. We present a challenging case with very atypical CNS and pulmonary presentations.

## Methods

We describe a case of a 9 yo girl who presented in April 2009 with vomits, headache, visual disturbance and ataxic gait of 1 month duration. A thorough investigation was performed, including 3 lumbar punctures and numerous microbiologic tests. A MRI revealed an infiltrative bulbar lesion suggestive of malignant glyoma. The lesion resolved after 30days with pulse therapy of methylprednisolone (MP) and intravenous immunoglobulin (IVIG). Three months later, the patient complained about recurrent painful oral ulcers and in April 2010, she presented with two genital similar lesions. She had new medical evaluation, diagnosing Behçet’s disease. In August 2010, the patient was admitted to the hospital with dyspnea, productive cough, fever and lung consolidation. She was treated with amoxicillin-clavulanate, with partial clinical response. Control chest X-ray showed a small atelectasis in the left lung base. In the following month, she was readmitted to the hospital complaining of intense dyspnea and chest pain, without fever. A new chest radiograph showed the same atelectasis and increased cardiac area. The echocardiogram revealed a large pericardial restrictive effusion. Pericardiocentesis and pericardial biopsy were performed and despite a wide etiology investigation, no evidence of microorganism was found. There was a complete clinical response with the increased dose of corticosteroid. One month later, she was readmitted to the hospital, presenting progressive dyspnea and cough. Admission chest X-ray showed a small consolidation in the right apex and that same atelectasis in the left base. A wide spectrum antibiotic regimen was prescribed, but the patient got worse, needing ICU, alveolar infiltrates became diffuse and oxygen saturation decreased. An open lung biopsy was performed and the patient developed respiratory failure, with acute respiratory distress syndrome, and needed mechanical ventilation and vasopressors use. A thorough investigation was again performed and the microbiological studies were negative. MP and IVIG were then administered, and the patient presented an excellent response, including chest radiograph normalization after 30 days. The lung biopsy revealed interstitial pneumonia.

## Results

After the whole case review, we were convinced that it is a severe case of Behçet’s disease, with atypical and life-threatening CNS and thoracic manifestations. This patient is now receiving cyclophosphamide pulse and tapering off prednisolone.

## Conclusion

More epidemiological data is necessary in pediatric Behçet’s disease to clarify the disease behavior and its peculiarities, enabling us to perform a more accurately diagnosis and a better management.

## Disclosure

Ana Beatriz Vargas: None; Cynthia T. França: None; Blanca E. R. G. Bica: None.

